# Liraglutide Dose Affordability and Durability in Real‐World Obesity Care

**DOI:** 10.1002/edm2.70364

**Published:** 2026-10-13

**Authors:** Azhar Zafar, Carina Scarlata

**Affiliations:** ^1^ Primary Care Research Hub Northampton UK; ^2^ NIHR Leicestershire and Northamptonshire Commercial Research Delivery Centre, Leicester Diabetes Centre Leicester UK; ^3^ University of Buckingham Medical School Buckingham UK

Dear Editor,

Tu and colleagues provide valuable prospective, multicentre post‐marketing evidence on liraglutide for adults with obesity in Taiwan. These findings suggest that clinically meaningful benefit may occur below 3.0 mg, supporting personalised rather than universal dose escalation [1].

The key implementation finding is that 49% of participants who did not reach 3.0 mg cited medication cost. Affordability is therefore a system‐level determinant of dose attainment, treatment continuity and real‐world effectiveness. Obesity care must consider whether a sustainable dose can be achieved and maintained, not simply whether treatment is effective at the target dose. Cost‐limited dosing risks inequitable treatment intensity based on ability to pay rather than clinical need. Interpretation requires caution. This single‐arm study excluded participants who withdrew before follow‐up; around half withdrew before week 13 and only 60 contributed to the week‐26 analysis. Results in continuing users are encouraging but cannot establish comparative effectiveness, discontinuation outcomes or long‐term weight maintenance. Randomised studies support liraglutide 3.0 mg with lifestyle intervention [2], while maintenance studies underline the need to consider persistence rather than short‐term loss alone [3].

A further question is whether lower‐dose success represents a reproducible, clinically appropriate strategy or an artefact of selection bias. In this surveillance cohort, lower exposure may have reflected four distinct mechanisms: intentional dose limitation due to gastrointestinal tolerability (clinically appropriate), cost constraints (a barrier to equitable care), clinical preference based on individual risk–benefit assessment or other unmeasured factors. Real‐world evidence increasingly documents the complex barriers to treatment persistence; discontinuation rates for GLP‐1 receptor agonists are substantial, with cost and tolerability emerging as principal drivers of dose escalation failure and early discontinuation [4].

The distinction between tolerability‐limited dosing and cost‐limited dosing is crucial for policy: the former represents appropriate personalised medicine, while the latter represents an avoidable barrier to equitable treatment. Analyses that stratify these mechanisms, relating achieved dose, duration, discontinuation reason and individual weight trajectory, would help distinguish tolerable, sustainable treatment from treatment limited by systemic financial barriers. Reporting denominators at each time point, including weight outcomes for those who discontinue where feasible, would make the real‐world picture more complete.

The implications extend beyond Taiwan. Although access pathways, reimbursement and service capacity differ between health systems, the affordability principle identified in Taiwan is directly applicable to NHS systems, where formulary restrictions and patient eligibility criteria similarly shape real‐world practice patterns. Both contexts illustrate how healthcare policy and commissioning decisions function as de facto clinical decision‐makers, determining which patients receive target doses and which receive cost‐rationed submaximal doses. Future pragmatic registries should therefore follow participants for 12–24 months, sufficient time to capture seasonal patterns, medication persistence trends and longer‐term weight trajectories, given that evidence indicates weight regain typically becomes apparent within 6–12 months following GLP‐1 discontinuation [5].

Registries should report dose exposure, persistence, discontinuation, outcomes, adverse events and barriers across socioeconomic and funding contexts. This will establish which submaximal doses are sustainable, for whom and how obesity care can deliver equitable, durable benefit.

## Author Contributions

**Azhar Zafar:** conceptualization, writing – original draft, resources, visualization, validation, methodology, software. **Carina Scarlata:** validation, writing – review and editing, project administration.

## Funding

The authors have nothing to report.

## Conflicts of Interest

The authors declare no conflicts of interest.

## Data Availability

Data sharing not applicable to this article as no datasets were generated or analysed during the current study.
